# Towards VDAC pharmacology: Developing small-peptide channel inhibitors inspired by nature

**DOI:** 10.64898/2026.10.13.758907

**Published:** 2026-10-19

**Authors:** Tatiana K. Rostovtseva, Philip A. Gurnev, David P. Hoogerheide, Sergey M. Bezrukov

## Abstract

The Voltage-Dependent Anion Channel (VDAC) forms a unique pathway across the mitochondrial outer membrane for small ions and water-soluble metabolites, such as ATP and ADP. Despite VDAC’s involvement in both normal regulation of respiration and in a wide variety of mitochondria-associated pathologies, there are, surprisingly, no known FDA-approved pharmacological agents targeting VDAC. Earlier, we have shown that VDAC can be regulated through its interactions with well-known cytosolic proteins – dimeric tubulin and α-synuclein (αSyn) – that are able to reversibly block this channel at nanomolar concentrations. We hypothesized that the architecture of a prospective VDAC inhibitor should fulfill two main requirements: possess a negatively charged disordered polypeptide C- or N-terminus as a pore blocking domain and feature a mitochondrial membrane-binding domain. Using αSyn as a template, we engineered three peptides and demonstrated that they all characteristically block VDAC reconstituted into planar lipid membranes in a controllable manner, thereby mimicking the actions of tubulin and αSyn. The VDAC blockage times induced by the synthetic peptides are comparable to αSyn-induced blockages, though the half-effect solution concentration for synthetic blockers is a hundred times higher than that of αSyn. Our work can be considered a proof-of-principle study that informs future development of mitochondria-targeting pharmacological tools.

